# Fit-cardiopulmonary resuscitation approach in public mass cardiopulmonary resuscitation teaching: A randomized control trial

**DOI:** 10.15537/smj.2023.44.5.20220941

**Published:** 2023-05

**Authors:** Amirudin Sanip, Mohd H. Isa, Azlan H. Abd Samat, Mohd J. Jaafar, Mohd R. Abdul Manaf, Meilya Silvalila, Ismail M. Saiboon

**Affiliations:** *From the Department of Emergency Medicine (Sanip, Isa, Abd Samat, Jaafar, Saiboon), Faculty of Medicine, Department of Public Health (Manaf), Faculty of Medicine, Universiti Kebangsaan Malaysia, Cheras, Kuala Lumpur, and from the Department of Emergency Medicine (Silvalila), Faculty of Medicine, Unversitas Syiah Kuala, Banda Aceh, Indonesia.*

**Keywords:** cardiopulmonary resuscitation, out-of-hospital cardiac arrest, exercise, simulation training, learning

## Abstract

**Objectives:**

To improve public awareness and the rate of bystander cardiopulmonary resuscitation (CPR), a novel and exciting approach called fit-CPR that incorporates mass CPR with high-intensity physical activity into the beat of locally favoured music was proposed. This study was conducted to measure the effectiveness of fit-CPR compared to the standard classroom method (CCM).

**Methods:**

Between 30th August to 29th November 2018, 129 participants from Syiah Kuala University, Banda Aceh, Indonesia, were randomized to learn CPR, either through fit-CPR or CCM protocol. All participants underwent pre, post, and 6-month retention tests. Each test had a 10-item questionnaire with CPR performance on a manikin that was assessed using a validated checklist.

**Results:**

Sixty-one (47.3%) participants completed the fit-CPR while 68 (52.7%) completed the CCM. There was a significant improvement in knowledge, performance, and quality of CPR from pre, post, and 6-month retention tests (*p*<0.01) in both groups. On high-quality CPR, the fit-CPR and CCM groups obtained an increased score of 285.0% and 151%, respectively, *p*=0.014 between pre and immediate post-test. Knowledge scores between fit-CPR and CCM groups showed an increase of 79.5% and 111.2%, respectively, *p*=0.002. Fit-CPR was completed between 52.5-57.5 minutes, while CCM took 75 minutes.

**Conclusion:**

The fit-CPR demonstrated a comparable outcome to standard CPR when teaching to the mass public with less time spent.

**B**ystander cardiopulmonary resuscitation (CPR) is a reliable way to enhance out-of-hospital cardiac arrest (OHCA) survival rates.^1^ It also reduces the likelihood of brain damage and nursing home admission.^2^ Although there is a lack of data on bystander CPR in Indonesia, the Pan-Asian Resuscitation Outcome Study (PAROS) highlights that only 39.3% of OCHA received bystander CPR in Asia-Pacific.^3,4^ Despite the improvement in OHCA survival, the public is unwilling to perform bystander CPR because of a lack of education, fear of disease transfer, legal liability, and concern about injuring the victims.^5^

A community-based study carried out in Jakarta showed only 39.6% of respondents had basic life support (BLS) training.^6^ Conventional methods of CPR teaching, such as CPR courses and training, pose a few challenges in terms of access to the public. These include high fees and a lack of understanding, enthusiasm, and time.^7,8^ One of the strategies to increase public participation is mass CPR education.^9^ This method has no set number, and researchers have noted the widespread CPR training involving hundreds to thousands of people.^9,10^

Non-communicable diseases such as heart disease and stroke account for the top 3 causes of death in Malaysia and Indonesia.^11,12^ Since healthy lifestyle activities minimise cardiovascular disease risk, the government and non-profit groups conducted almost weekly physical activities to involve the public, like running, cycling, and aerobics.^13,14^

Based on the literature, an interesting approach of teaching mass CPR combined with a moderate-intensity fitness activity; hence, the name “fit-CPR” was introduced. The program was first conducted in Malaysia and was favourably welcomed. The entire event was carried out in Malay, using the 5-Ts abbreviation for hands-only CPR.^15^ As Malay is the main language of the Malay archipelago (*Nusantara*), we sought to examine the efficiency of this unique strategy on a group of laypeople in Indonesia who also speak the same language.

## Methods

This was a prospective randomized controlled trial to compare the effectiveness of fit-CPR (intervention) against the conventional classroom method (CCM) in teaching hands-only CPR (control). This study was carried out in Banda Aceh, Indonesia, from August to November 2018. It involved Syiah Kuala University, Aceh, Indonesia students. We received approval from the Universiti Kebangsaan Malaysia (UKM), Medical Research and Ethics Committees and carried out this study following the Helsinki Declaration. This study includes pre-test, post-test, and 6-month retention tests. The Ethical approval was obtained from the Research Ethics Committee of the Faculty of Medicine, Universiti Kebangsaan Malaysia (UKM FF-2018-345).

The study was carried out using convenience sampling during the orientation week for new university students. An announcement on the study was carried out, and those who were interested voluntarily join the study. Those who had CPR training within the previous 2 years, not physically healthy or refused to participate were excluded. Those who did not finish the study protocol were excluded. They received details regarding the study’s process and were given a consent form. Name, age, gender, comorbidities, contact information, experience learning or doing CPR, and degree of physical activity were all recorded as necessary.

**Figure 1:**
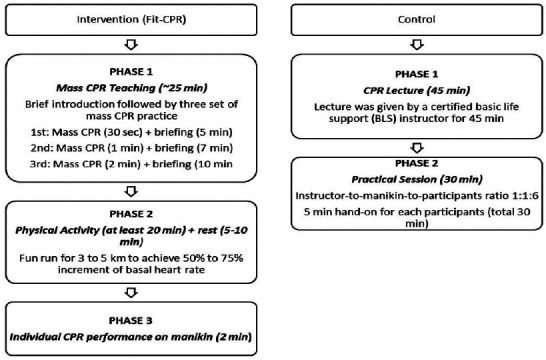
- Flow diagram of the intervention group versus the control group. CPR: cardiopulmonary resuscitation, min: minutes

The intervention was divided into 3 phases: i) 25 minutes (mins) of hands-only CPR teaching accompanied by music, ii) 20 mins of moderate-intensity physical activity, and iii) 2 mins of hands-only CPR on a manikin (Figure 1). The CPR applied the new 5-T steps approach: Tengok, Tegur, Teriak @ Telefon, Teliti, and Tekan, which are Malay phrases that translate as Look, Check (for answer), Shout @ Call, Assess, and Compress.

The first phase started with the main instructor introducing the fit-CPR protocol. Following that, 3 rounds of mass CPR practice were conducted with music and interspersed with a mini-briefing. Each round involved CPR practice and mini-briefings (first round: 30 seconds CPR practice and 5-minute mini-briefing; second round: 1-minute CPR practice and 7-minute mini-briefing, and the third round: 2-minute CPR practice and 10-minute mini-briefing plus a question and answer ([Q&A] session). The longer mini-briefing session allowed for recovery and more participant inquiries. The briefings were kept short to avoid boredom while focusing on performing CPR’s critical knowledge and foundations. Attendees learned on the 5-T CPR principles, indications, contraindications, and CPR termination during this mini-briefing and Q&A session. The participant-to-manikin ratio was kept at 1:1. The instructors’ role was to circulate among the participants, connect with them, answer questions, and teach hands-only CPR to the beat of the music. Demonstrators were positioned in front of the attendees on an elevated platform to facilitate the practice while-watching method. The facilitators moved among the participants, assisting them in performing CPR using only their hands (hands-only CPR).

The second phase involved physical activity, which included running for at least 20 mins on a designated pathway, followed by a 5- to 10-mins rest interval. Participants were expected to increase their basal heart rate by at least 60% during this activity. The third phase required participants to execute hands-only CPR within 2 mins without the assistance of instructors, facilitators, demonstrators, or any music.

Participants in the control group were required to complete the following 2 phases: i) a 45-minute lecture on cardiopulmonary resuscitation (delivered by a certified American Heart Association basic life support instructor). The lecture discussed the science and concepts of cardiopulmonary resuscitation, its indications and contraindications, and the 5 CPR steps of Danger, Response, Airway, Breathing, and Circulation (DRABC); ii) 30-mins face-to-face classroom teaching hands-only CPR with a ratio of 1:1:6 instructor-to-manikin-to-student.

As a means of measurement, a validated questionnaire and Objective Structured Clinical Examination (OSCE) checklist were used to assess participants’ basic CPR knowledge and performance, which had been obtained from the author of a previously published study.^16^

The outcomes measured for CPR knowledge were as follows: CPR indication, correct steps of hands-only CPR, the emergency number, hand placement, compression rate, compression depth, and CPR termination. The performance of CPR was evaluated using the OSCE checklist, which included questions about the victim’s response, calling for help, the steps of CPR, hand placement, and performing high-quality CPR. High-quality CPR involved a push-fast (100 to 120 chest compressions per minute), push-hard (at least 2-inch depth or a click sound on the mannequin), and adequate chest recoil with minimal interruption of chest compression.

The sample size was calculated using OpenEpi (JavaScript, MIT), which is an online open-source application.^17^ The desired confidence interval of 95%, power of 80%, and the ratio of the sample sizes of groups 1 and 2 (1:1) were set. We used a previous study comparing 2 groups in terms of intervention and control.^18^ We used a mean of 6.85 (standard deviation [SD], 1.21) for group 1 and 6.2 (SD, 1.04) for group 2. The minimum total sample size for both groups was 96 participants.

The participants were divided into intervention and control groups using Research Randomizer (Urbaniak, G. C., & Plous, S. [2013)]). The researcher randomly assigned participants to both groups via concealment of allocation. Each participant was assigned a unique code (UC) number to avoid bias. Throughout the trial, they could not change their UC number. The UC was 3 digits. An intervention participant’s code was 1XX, while a control participant’s code was 2XX. The first digit corresponded to the intervention/control group, while the second and third were the participant numbers. For example, UC ‘201’ was the first participant in the control group, and UC ‘108’ was the eighth person in the intervention group.

Emergency physicians, senior paramedics, medical officers, and medical educators volunteered as assessors. To limit inter-rater discrepancies, a video calibration workshop and a debriefing session were organized. Assessors watched 5 videos of various CPR performances and scored them using the checklist during the video calibration session. Recalibration was performed until the assessors’ scores for a particular performance diverged by not more than one. The assessors were debriefed in order to improve the study’s comprehension and scoring process. The assessors were blinded to the intervention and control groups during the CPR sessions.

### Statistical analysis

All data were analyzed using IBM SPSS for Windows (version 23, IBM Corp., Armonk, N.Y., USA). The normality test was performed using Kolmogorov–Smirnov.^19^ We used a student paired T test for continuous variables (knowledge and performance) and a Chi-square test for categorical variables (demographic data). Continuous variables were presented as means±SD and categorical variables as numbers and percentages. The general linear model (GLM) was used to compare the mean between the groups with Bonferroni correction. The 2-tailed significance level of *p*<0.05 was implemented.

## Results

**Figure 2:**
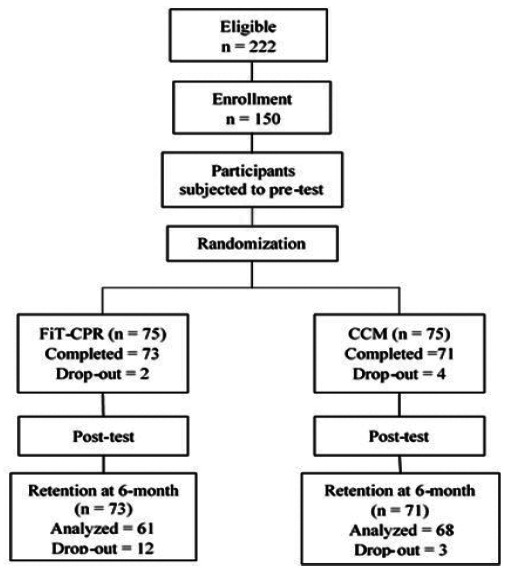
- Consort flow diagram of the study. CCM: conventional classroom method, CPR: cardiopulmonary resuscitation

Hundred and fifty out of 222 eligible people signed up for the study. Each group had 75 participants. However, 21 people dropped out of the study by virtue of not attending the programs. Out of 129 participants, 61 (47.3%) were in the intervention group, and 68 (52.7%) were in the control group (Figure 2). The age, gender, degree of physical activity, and number of CPR courses attended in the last 5 years were not significantly different between the 2 groups (*p*>0.05) (Table 1).

**Table 1:** - Demographic data of both fit-CPR and CCM programs.

Group	Fit-CPR (n = 61)	CCM (n = 68)	*p*-value
***Age***			
17 years old	61 (100)	68 (100)	*p*>0.05
***Gender***			
Male	19 (31.1)	22 (35.3)	*p*=0.709
Female	42 (68.9)	44 (64.7)	
***Level of physical activity***			
<1×/week	25 (41.0)	33 (48.5)	*p*=0.120
1×/week	24 (39.3)	23 (33.8)	
2–3×/week	11 (18.0)	6 (8.8)	
>3×/week	1 (1.6)	6 (8.8)	
***Number of CPR courses attended in 5 years***			
0	59 (96.7)	66 (97.1)	*p*=0.259
1	2 (3.3)	0 (0.0)	
2	0 (0.0)	1 (1.5)	
>2	0 (0.0)	1 (1.5)	
***Background medical illness***			
Yes	0 (0)	0 (0)	*p*>0.05
No	61 (100)	68 (100)	
***Actively perform CPR within 2 years***			
Yes	0 (0)	0 (0)	*p*>0.05
No	61 (100)	68 (100)	

Values are presented as numbers and percentages (%). CCM: conventional classroom method, CPR: cardiopulmonary resuscitation

The intervention group utilized 8 trainers (2 instructors, 2 demonstrators, and 4 facilitators) to complete the program, whereas the control group used 13 (1 lecturer and 12 facilitators). The intervention group took 52.5–57.5 mins to complete the 3 phases (teaching, running, resting, and performing CPR), while the control group took 75 mins (lecture and CPR training).

**Figure 3:**
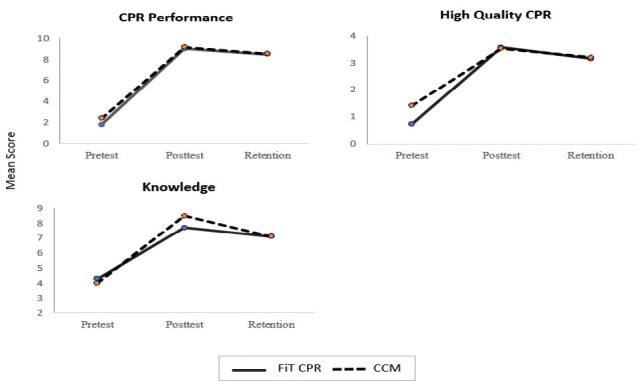
- Graph comparison of mean scores for cardiopulmonary resuscitation (CPR) performance, high-quality CPR and knowledge, between the Fit-CPR program (solid lines) and CCM (dashed lines). CCM: conventional classroom method

There were significant increments and decrements in knowledge scores for pre-test to post-test and post-test to 6-month retention test, respectively, in both groups (general linear model [GLM] within-subject effect, *p*<0.001). The knowledge score for the intervention group increased by 79.5% at the post-test and decreased by 8% at the 6-month retention test, whereas the score increased by 111.2% at the post-test and decreased by 16% at the 6-month retention test for the control group (Figure 3). There was a significant difference in scores between both the groups, wherein the control group was better than the intervention group (GLM between-subject effects, *p*=0.002) (Table 2).

**Table 2:** - Comparison of mean scores for knowledge, performance, and high-quality cardiopulmonary resuscitation (CPR).

Items evaluated	Fit-CPR n=61	CCM n=68	GLM within-subject effect (*p*-value) ^a^	GLM between subject effect (*p*-value) ^a^
			Mean (SD)	
***Knowledge***				
Pre-test	4.34 (1.40)	4.03 (1.40)	<0.05*	0.002*
Post-test	7.79 (1.31)	8.51 (1.03)		
Retention	7.16 (1.33)	7.15 (1.65)		
***Performance***				
Pre-test	1.80 (1.41)	2.41 (1.72)	<0.05*	0.261
Post-test	9.02 (0.98)	9.18 (1.17)		
Retention	8.49 (1.21)	8.54 (1.73)		
***High-quality CPR***				
Pre-test	0.72 (1.10)	1.41 (1.34)	<0.05*	0.014*
Post-test	3.57 (0.67)	3.54 (0.78)		
Retention	3.16 (0.90)	3.21 (0.86)		

^a^ Comparison of trends between the 2 groups was analyzed using the general linear model (time and group interaction effect).

^*^ Statistically significant results are *p*<0.05. CCM: conventional classroom method, GLM: general linear model

In the knowledge sub-group, a significant improvement was seen at the immediate post-test for the intervention group in the indication of CPR (*p*=0.001), and the control group had better knowledge of the rate and depth of chest compression (*p*<0.001) and CPR termination (*p*=0.004). The remaining items showed no significant difference (Table 3).

**Table 3:** - Knowledge scores for the intervention and control groups.

Group	Fit-CPR (n=61)	CCM (n=68)	*P*-value
	Correct answer n (%) Mean±SD		
***Indication (Q1)***			
Pre-test	7 (11.5)	11 (16.2)	0.442
Post-test	39 (63.9)	24 (35.3)	0.001
Retention	22 (36.1)	14 (20.6)	0.050
***CPR Steps (Q2, Q3, Q5, Q6)***			
Pre-test	2.03±0.93	1.99±0.99	0.779
Post-test	3.80±0.44)	3.85±0.36	0.481
Retention	3.61±0.53	3.51±0.68	0.396
***Emergency number (Q4)***			
Pre-test	54 (88.5)	56 (82.4)	0.323
Post-test	61 (100.0)	68 (100.0)	1.000
Retention	59 (96.7)	64 (94.1)	0.483
***Hand placement (Q7)***			
Pre-test	44 (72.1)	51 (75.0)	0.712
Post-test	56 (91.8)	62 (91.2)	0.899
Retention	49 (80.3)	59 (86.8)	0.323
***Rate & depth (Q8, Q9)***			
Pre-test	0.57±0.76	0.28±0.54	0.012
Post-test	1.03±0.97	1.75±0.61	0.000
Retention	1.16±0.86	1.29±0.87	0.394
***CPR termination (Q10)***			
Pre-test	1 (1.6)	2 (2.9)	0.624
Post-test	24 (39.3)	44 (64.7)	0.004
Retention	16 (26.2)	22 (32.4)	0.446

CCM: conventional classroom method, CPR: cardiopulmonary resuscitation

The scores for CPR performance also showed significant changes in scores between the pre-test, post-test, and 6-month retention tests for both groups (GLM within-subject effect, *p*<0.001). The intervention group showed a 401.1% score increment while the control group showed a 280.9% score increment in the post-test. During the 6-month retention test, the intervention group showed a 5.9% score decrement, whereas the control group showed a 7% score decrement (Figure 3). However, there was no significant difference in the scores between the groups (GLM between-subject effects, *p*=0.261) (Table 2).

Measurement of high-quality CPR components (compression depth, rate, recoil, and minimal interruption) showed that the score of the intervention group increased by 285%, while the control group increased by 151% at the post-test. At the 6-month retention test scores, the intervention group showed a decrease of 11.5%, while the control group recorded a decreased by 9.3%. The trends were significantly different in both groups (GLM within-subject effects, *p*<0.001) (Figure 3). However, there was a significant difference in the scores between the 2 groups, wherein the intervention group was better than the control group (GLM between-subject effects, *p*=0.014) (Table 2).

## Discussion

In this study, the fit-CPR approach showed a significant improvement from baseline to retention, especially in CPR performance and high-quality CPR with less time (57.5 versus 75 mins) and fewer facilitators (6 versus 13).

We had more female participants, as female students outnumber male students in most of Southeast Asia, especially Indonesia.^20^ The results showed that the intervention group could perform hands-only CPR remarkably well and was comparable to the control group in terms of CPR knowledge and performance. It is interesting to note that the baseline performance of the intervention group at the pre-test was significantly inferior compared to that of the control group. After learning hands-only CPR, both groups performed equally well, with no significant differences in performance during the immediate post-test. It showed that an effective technique could improve the participant’s performance even with poor baseline performance. It also used fewer facilitators and a shorter training period than the control group did. Although both groups’ performance scores decreased during the retention stage, their performance were still significantly better than in the pre-test.

Many factors, such as a simpler approach to using the 5-T, memory-enhancing physical activity, or music influence, could explain the intervention group’s comparable performance to the control group despite shorter training time and fewer facilitators.^21-25^ Physical activities have been shown to improve knowledge and skill acquisition by producing more brain-derived neurotrophic factor (BDNF), which is essential for long-term memory, and lactate, which is essential to the brain’s energy supply.^22,26^ Increment of BDNF and lactate are more pronounced with moderate to vigorous-intensity exercise (increases 60%-80% of baseline heart rate) lasting for 20 to 40 mins.^21^ It is important since high lactate levels increase motor cortex excitability.^26^ The accessibility of lactate has an important influence on long-term memory formation, as the expression of monocarboxylate-transporter (MCT) causes a decrease in transferring lactate to astrocytes and neurons in vitro, which results in compromised long-term memory.^23^

In terms of knowledge, there was no significant difference between both groups in the pre-test, but the control group significantly showed better knowledge acquisition in the post-test compared to the intervention group. This could be because the control group received more theoretical instruction (45 mins) than the intervention group (20 mins). It showed a clear advantage of conventional CPR teaching that separates the theory and practical aspects of CPR.^27^ However, in the sub-group analysis, the intervention group showed better knowledge of the sign of CPR. In the intervention group, facilitators provided several scenarios for performing CPR, which may have contributed to this outcome. This aspect was deficient in the control group, where there was only one slide regarding the indication of CPR. Therefore, it was evident that learning by scenario (experiential learning) had better results compared to didactic lectures.^28^

The control group showed better knowledge of CPR termination and the components of high-quality CPR, which were the rate and depth of chest compression. Knowledge of high-quality CPR that involved numbers like a compression rate of 100–120 per min, compression depth of 5–6 cm or 2 inches, and minimal interruption of fewer than 10 seconds were all better understood through didactic lectures. This might be explained by the utilisation of visual and auditory stimulation in the control group, which enhanced memory, in contrast to the intervention group who learned only through auditory stimulation.^29,30^

Language also plays a role in learning. Graham and Guy believe that teaching students in their native language will aid in their learning. In terms of retention, the intervention group, which was exposed to the CPR steps using the 5-T acronym (Malay version of DRABC), showed better retention compared to the control group using DRABC, although this was not statistically significant. The acronyms could facilitate learning and memorizations.^31,32^ Using a novel 5-T acronym in the native language may synergize this technique’s efficacy even though the contact time is short. In Japan, where English is not a native language, CPR is taught in the Japanese language, leading to high awareness and bystander CPR rates.^33^ It is an exciting aspect that can be further investigated.

The findings from the present study provide valuable insights towards the development of alternate, efficient CPR training program that considers memory-enhancing techniques and linguistic factors. Further research could investigate the generalizability of these findings to other populations and settings.

### Study limitation

First, CPR performance was assessed by an individual assessor with no objective measurement of the depth, rate, and chest recoil. A more objective CPR assessment device would improve accuracy and reliability.^34^ Second, despite the careful calibration of the assessors, we did not objectively measure inter-rater variability. Therefore, the inter-class coefficient could not be generated. Third, we did not assess the participants’ BDNF levels because of logistical issues. However, in our future study, measuring the BDNF level with different levels of exercise intensity will provide a better understanding of its role. Finally, the study was carried out in one location. In the same country, findings may be comparable or different. The samples had the same makeup and characteristics, which may limit the result’s generalizability.

In conclusion, fit-CPR, an innovative technique of teaching CPR to laypeople, produced comparable outcomes to standard CPR teaching. This method significantly improved CPR performance, which was initially low. This new method enable more participants to be taught more efficiently in shorter duration and lesser facilitators. This method used music, engaging instructor–participant interaction, and physical activities to make learning CPR fun. This method may be repeated and developed to teach lay people CPR, thus increasing bystander CPR rates.
